# Long Non-Coding RNAs and Their Potential Roles in the Vector–Host–Pathogen Triad

**DOI:** 10.3390/life11010056

**Published:** 2021-01-14

**Authors:** Parwez Ahmad, Chaima Bensaoud, Imen Mekki, Mujeeb Ur Rehman, Michail Kotsyfakis

**Affiliations:** 1 Institute of Parasitology, Biology Centre, Czech Academy of Sciences, 37005 Ceske Budejovice (Budweis), Czech Republic; ahmad.parwez@gmail.com (P.A.); bensaoud.chaima@gmail.com (C.B.); makkiimene@gmail.com (I.M.); 2 Faculty of Science, University of South Bohemia, 37005 Ceske Budejovice, Czech Republic; 3 Key Laboratory of Animal Disease and Human Health of Sichuan Province, Sichuan Agricultural University, Wenjiang, Chengdu 611130, China; mujeebnasar@yahoo.com

**Keywords:** ncRNA, lncRNA, vector–host–pathogen interactions

## Abstract

Long non-coding (lnc)RNAs have emerged as critical regulators of gene expression and are involved in almost every cellular process. They can bind to other molecules including DNA, proteins, or even other RNA types such messenger RNA or small RNAs. LncRNAs are typically expressed at much lower levels than mRNA, and their expression is often restricted to tissue- or time-specific developmental stages. They are also involved in several inter-species interactions, including vector–host–pathogen interactions, where they can be either vector/host-derived or encoded by pathogens. In these interactions, they function via multiple mechanisms including regulating pathogen growth and replication or via cell-autonomous antimicrobial defense mechanisms. Recent advances suggest that characterizing lncRNAs and their targets in different species may hold the key to understanding the role of this class of non-coding RNA in interspecies crosstalk. In this review, we present a general overview of recent studies related to lncRNA-related regulation of gene expression as well as their possible involvement in regulating vector–host–pathogen interactions.

## 1. Introduction

**Figure 1:**
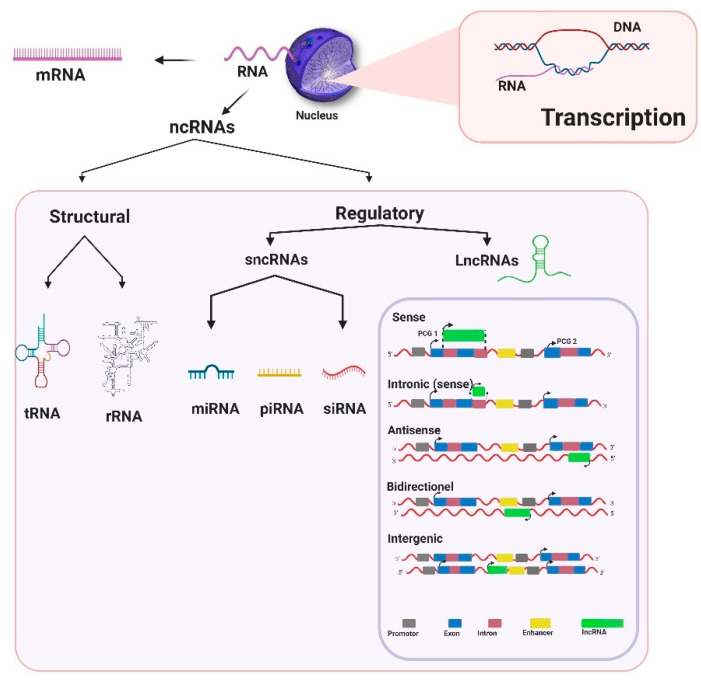
Classification of non-coding RNAs (ncRNAs). In the nucleus, DNA is transcribed into two main RNA transcripts: messenger RNA (mRNA), which is translated into proteins, and ncRNAs, which perform regulatory functions. ncRNAs can be classified into structural and regulatory classes. The structural class includes ribosomal RNA (rRNA) and transfer RNA (tRNA), while the regulatory class can be divided into two main categories, namely small non-coding RNAs (sncRNAs) and long non-coding RNAs (lncRNAs). SncRNAs include microRNAs (miRNAs), piwi-interacting RNAs (piRNAs), and endogenous small-interfering RNAs (siRNAs). LncRNAs can be classified according to their position and the nearest protein-coding genes (PCGs): sense lncRNAs are located within a PCG and span multiple introns or exons; intronic lncRNAs are located on the sense strand within an intron of coding genes; antisense lncRNAs are located on the opposite strand of a PCG; bidirectional lncRNAs are transcribed from the opposite strand within 1 kb of the promoter of the sense strand; and intergenic lncRNAs, which are always located between two PCGs.

Ribonucleic acids (RNAs) play an intrinsic role in the origin of life through both their genetic and catalytic properties [1]. According to the “RNA world hypothesis”, early life started with self-replicating RNA molecules prior to the evolution of DNA and proteins [2]. In humans, only 2% of RNA transcripts are translated into proteins [3], and for a long time, the remaining proportion was thought to be devoted to housekeeping functions. As our understanding of the genome advanced in tandem with genomic technology developments, it is now clear that transcripts lacking protein-coding potential possess diverse regulatory functions; these RNAs are known as non-coding RNAs (ncRNAs) [4] and can be classified into structural and regulatory ncRNAs [5]. Structural ncRNAs include transfer RNA (tRNA) and ribosomal RNA (rRNA). rRNAs form a primary component of ribosomes as both large and small subunits in prokaryotic and eukaryotic cells [5]. tRNAs are 76–90 nucleotide (nt) RNAs that play an important role in linking mRNAs and amino acid sequences at ribosomes during protein synthesis [6]. Regulatory ncRNAs can be divided into two main types based on their size, namely small non-coding RNAs (sncRNAs) <200 nt long and long non-coding RNAs (lncRNAs) >200 nt long [7,8] (Figure 1). A number of small regulatory RNA types have now been defined, with microRNAs (miRNAs), piwi-interacting RNAs (piRNAs), and endogenous small-interfering RNAs (siRNAs) being the most thoroughly investigated classes [9].

LncRNAs, the focus of this paper, are the most prevalent and functionally diverse class of RNAs [10]. Most lncRNAs share traits with mRNA such as alternative splicing and transcriptional control [11]. However, lncRNAs are usually transcribed from fewer exons than coding RNAs and lack an open reading frame [12,13,14]. They mostly locate to the nucleus, where they occupy various nuclear compartments such as the nucleoplasm, sub-nuclear domains, and chromatin. Once localized in the nucleus, they can act as key regulators of nuclear organization and function [13]. Some lncRNAs are also found in the cytoplasm or in both the cytoplasm and nucleus [15,16]. LncRNAs can be classified into five categories according to their relative position to their nearest protein coding genes (PCGs) [17,18,19]: (i) sense lncRNAs, spanning multiple introns or exons within a PCG; (ii) intronic lncRNAs, located within an intron of a coding gene on the sense strand; (iii) antisense lncRNAs, which are transcribed from the opposite strand of a PCG; (iv) bidirectional lncRNAs, situated on the opposite strand but within 1 kb of the promoter on the sense strand (Figure 1) and transcribed in the direction opposite to that of the promoter on the sense strand; and (v) intergenic lncRNAs, which are transcripts located between two PCGs [20].

Emerging evidence supported by cutting-edge sequencing technologies and annotation tools have shed light on lncRNA functions related to transcriptional and post-transcriptional control, i.e., DNA methylation, histone modification, splicing, transcription, and translation [2]. Given these diverse roles, lncRNAs also regulate several cellular phenotypes including cellular differentiation and malignant transformation [21]. Despite the established importance of lncRNAs, investigations into their involvement in interspecies cross-talk, especially with respect to the pathogenesis of vector-borne infections, are still in their infancy. There is empirical evidence that lncRNAs can either be vector-derived, host-derived, or pathogen-encoded. Some lncRNAs enhance pathogen survival, and some help to enhance the host immune defense system [22,23].

In this review, we describe recently discovered lncRNAs implicated in gene expression regulation in the context of vector–pathogen–host interactions. We also highlight challenges in the field and future research perspectives.

## 2. Structure and Biogenesis of lncRNAs

LncRNAs are a poorly structurally characterized class of RNAs, since existing analytical techniques are challenging and ill-suited for lncRNA studies, needing appropriate experimental systems, which should allow a better understanding of the functions of lncRNA, and their roles in both physiological and pathological conditions [24]. LncRNAs can interact with DNA, RNA, or proteins to control epigenetic, transcriptional, or translational responses [2]. In contrast to coding genes, the primary transcribed gene sequence of lncRNAs tends to show low levels of homology across species [25], but lncRNAs from related species may have particularly well-conserved secondary or tertiary structures in structural motifs (Figure 1). Consequently, lncRNA-related gene regulatory roles may be preserved despite a lack of primary sequence conservation [26].

**Figure 2:**
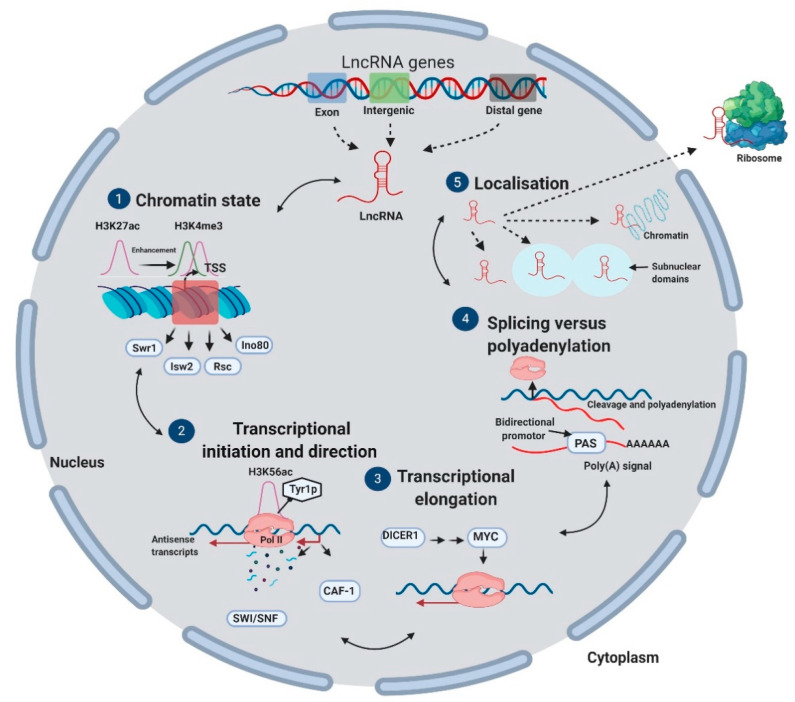
LncRNA biogenesis. Long non-coding RNAs can be transcribed from exonic, intergenic, or distal coding regions of DNA. They are regulated according to their biogenesis timepoint. (1) Throughout maturation, at the chromatin level, lncRNAs exhibit enrichment of H3K4me3 at promoters and enrichment of H3K27ac repressed by chromatin remodeling complexes (Swr1, Isw2, Rsc, and Ino80). (2) At the transcriptional level, initiation is in the divergent antisense direction. Transcription is enriched for H3K56ac and phosphorylation of RNA polymerase II (Pol II) Tyr1. SWI/SNF proteins enhance transcription in the divergent direction, and CAF-1 represses it. (3) Transcriptional elongation is regulated mainly by DICER1 and MYC. (4) The splicing of mRNA is in the sense direction; however, its cleavage and polyadenylation are in the divergent antisense direction. (5) The last level of lncRNA maturation is localization: lncRNAs can localize to chromatin, subnuclear domains, and the nucleoplasm, while certain lncRNAs leave the nucleus to localize to ribosomes.

The biogenesis and origin of lncRNAs are incompletely understood. However, recent studies have proposed that over two thirds of lncRNA transcripts may contain transposable elements (TEs) [27,28], suggesting that they form via the insertion of TEs into the genome [27,28]. In mammals, lncRNAs can be transcribed from intergenic, exonic, or the distal protein-coding regions of the genome by RNA polymerase II (Figure 2). At the chromatin level, lncRNAs and mRNAs exhibit similar properties such as an enrichment of H3K4me3 (trimethylated histone 3 at lysine 4) at promoters. However, lncRNA genes have higher enrichment of H3K27 (trimethylated histone 3 at lysine 27) and are more strongly repressed by certain chromatin remodeling complexes such as Swr1, Isw2, Rsc, and Ino80 (Figure 2). LncRNA transcription initiates from divergent promoters according to the RNA direction, with many described lncRNAs transcribed antisense from coding gene promoters. Transcription in the divergent direction is further enhanced by SWItch/sucrose non-fermentable (SWI/SNF) complex proteins and repressed by chromatin assembly factor (CAF)-1. The occurrence of U1 and polyadenylation signals differs on either side of bidirectional promoters, favoring the splicing of mRNAs in the sense direction and their cleavage and polyadenylation in the divergent, antisense direction (Figure 2). Compared to RNAs, lncRNAs are more variably localized, as certain lncRNAs occupy the chromatin, subnuclear domains, nucleoplasm, or cytoplasm, while mRNAs localize very specifically to ribosomes in the cytoplasm [5].

## 3. LncRNA Mechanisms of Action

**Figure 3:**
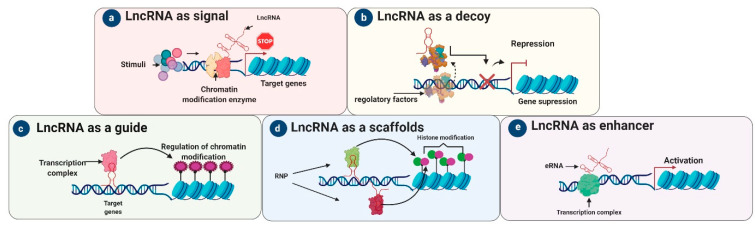
Archetypes and mechanisms of action of lncRNAs. LncRNAs execute five main molecular functions depending on their mechanism of action. (**a**) Signal lncRNAs: responding to a stimulus, lncRNAs receive the signal to interact with chromatin-modifying enzymes to prevent transcription. (**b**) Decoy lncRNAs have more affinity for particular regulatory factors; once bound, they lead to transcriptional repression by preventing these regulatory factors from binding to the DNA. (**c**) Guide lncRNAs arrange transcription factors at specific genomic loci and help to regulate chromatin. (**d**) Scaffold lncRNAs help to assemble RNA-protein (RNP) complexes; their role is to promote or suppress transcription by activating or repressing target genes. (**e**) Chromatin interactions are influenced by an enhancer that can be presented by an enhancer RNA (eRNA).

LncRNAs are therefore a diverse group of regulatory ncRNAs with various characteristics, localizations, and modes of action [29]. A comprehensive classification of lncRNAs is quite challenging, as most do not share structural, functional, or mechanistic features. Moreover, only a few lncRNAs have been thoroughly mechanistically characterized to date, with even fewer being functionally verified in vivo [30]. Overall, their functions depend on their subcellular localization, i.e., in the cytoplasm or nucleus. Here we describe the five archetypal molecular functions of lncRNAs: as signals, decoys, guides, scaffolds, and enhancers (Figure 3).

### 3.1. LncRNAs as Signals

Signal lncRNAs are expressed at a specific time and in a specific position in the cell in response to stimuli. Some lncRNAs in this archetype are regulatory, while others are merely by-products of transcription, with the act of initiation, elongation, or termination being regulatory. Signal lncRNAs (Figure 3) are known to interact with chromatin-modifying enzymes such as histone methyltransferases to silence their target genes by blocking their transcription or through heterochromatin formation [16].

### 3.2. LncRNA as Decoys

“Decoy” lncRNAs can act as molecular sinks by decreasing the accessibility of particular regulatory factors via decoy binding sites [16]. These lncRNAs repress transcription, in an indirect way, by sequestering regulatory factors (Figure 3) including miRNAs, modifying complexes (chromatin subunits), catalytic proteins, and transcription factors, reducing their availability [16,31]. LncRNA decoys negatively modulated transcription by not allowing a particular effector to interact with its intrinsic target [16]. For example, the decoy lncRNA PANDA regulates apoptosis [16]: NF-YA is a transcription factor responsible for the activation of apoptosis-related genes, but when PANDA binds to NF-YA it is sequestered away from its target genes, resulting in the suppression of apoptosis-related genes [32,33]. Other decoy lncRNAs like *TUG1* and *MEG3* indirectly degrade and alter protein translation by isolating miRNA from mRNA and protein targets [16,34,35]. In addition, many other lncRNAs, such as highly up-regulated in liver cancer (*HULC*), H19, growth arrest-specific 5 (*GAS5*), *HOTAIR*, phosphatase and tensin homolog pseudogene 1 (*PTENP1*), and *MALAT1,* act as decoys by preventing transcription factors or miRNA from binding with their target sites, thereby regulating translation and transcription (Figure 3).

### 3.3. LncRNA as Guides

To regulate the genome, guide lncRNAs are necessary for the organization/localization of factors at specific genomic loci. As such, lncRNA guides direct chromatin modifiers and protein complexes (such as RNPs) or transcription factors to particular target gene(s) and help them localize appropriately at their transcriptional loci [36]. The exact targets of guide lncRNAs (Figure 3) are stimulated by RNA–RNA, RNA–protein, and RNA–DNA interactions. However, the exact details of this mechanism are uncertain [37]. LncRNAs such as *HOTAIR*, functional intergenic repeating RNA element (*FIRRE*), *COLDAIR*, *KCNQ1* overlapping transcript 1 (*KCNQ1OT1*), taurine-upregulated gene 1 (*TUG1*), *HOTTIP*, *XIST*, *MEG3*, and *ANRIL* can act as guides by reprograming chromatin complexes and managing the recruitment of epigenetic modifiers to their definitive loci [38]. For instance, *KCNQ1OT1* and *HOTAIR* bind to PRC2, a chromatin modifier, to regulate target genes in cis or trans and thereby inhibit gene expression [39,40,41].

### 3.4. LncRNA as Scaffolds

Scaffold lncRNAs play an important structural role in assembling multi-protein complexes such as short-lived ribonucleoprotein (RNP) complexes [42]. After the complete assembly of RNP complexes, they can suppress or activate transcription depending on the existence and nature of the involved RNAs and proteins [42]. For example, the well-studied lncRNA *TERC* is an excellent example of a molecular scaffold, in which the telomerase complex combines associated proteins with reverse transcriptase action in a single RNP [43]. *TERC* is a more stable molecular scaffold lncRNA than all other newly identified lncRNAs (Figure 3). As substitutes, lncRNAs might have low-affinity interactions with protein-like mRNAs as they mature. In addition, lncRNAs like *MALAT1*, *ANRIL*, and *TUG1* can serve as dynamic scaffolds by associating with PRC1 and PRC2 to stimulate target gene suppression or activation [31,44].

### 3.5. LncRNA as Enhancers

In the signal archetype, the lncRNA sends a molecular signal to initiate regulation at the transcriptional level in response to different stimuli [35,45]. For instance, *KCNQ1OT1* recruits PRC2 and chromatin-modifying enzymes (histone methyltransferases) by acting as a signal lncRNA [46]. In the enhancer archetype (Figure 3), the “chromatin interaction” of DNA is influenced by the enhancer regions (ERs) as a result of enhancer RNAs (eRNAs). It is thought that these lncRNAs are not released from ERs but tether proteins to ERs [47].

Finally, lncRNAs may have additional regulatory functions such as trafficking and protein signaling, which require further study.

## 4. LncRNAs in Vector–Host–Pathogen Interactions

**Figure 4:**
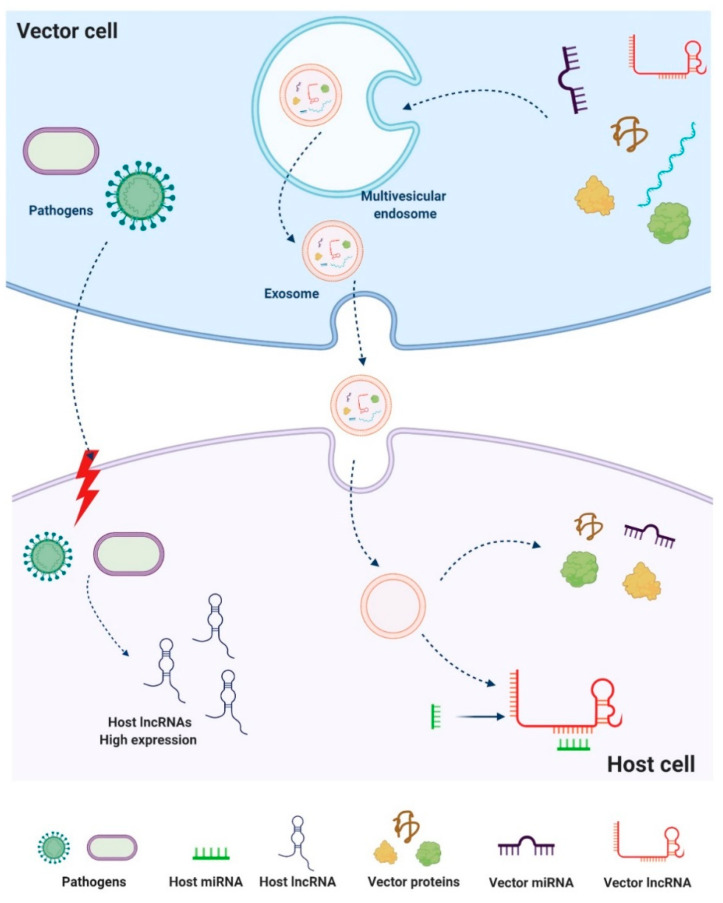
Proposed model for regulatory networks mediated by lncRNAs in vector–host–pathogen interactions. LncRNAs might be secreted within exosomes, membrane-containing vesicles, from vector cells. They are sorted and excreted by exosomes in the host cell, thereby facilitating efficient vector–host interactions. Inside the host cell, vector lncRNAs may act as regulators of the host genomic output, disturbing host homeostatic signaling pathways.

Arthropod vectors are of veterinary and medical importance, as they can transmit pathogens to cause diseases in animals and humans [48]. Many of these vectors are bloodsucking arthropods, which ingest pathogens during a blood meal from an infected host and later inject it into a new host during their subsequent blood meal [49]. While feeding, vectors transmit pathogens and inject, through their saliva, a cocktail of bioactive molecules into their vertebrate hosts to subvert host defense responses [50]. Understanding how these blood-feeding arthropods transmit pathogens requires knowledge of the molecular and cellular interplay at the vector–host interface. Recent reviews have highlighted the role of sncRNAs in trans-kingdom and inter-species communication in the context of vector–host–pathogen interactions [51,52]. However, few studies have focused on the roles of lncRNAs in this network. LncRNAs, like lipids, proteins, DNA, and mRNA, are transported in vertebrates via biological carriers called exosomes [53]. Exosomes, a type of extracellular vesicle (EV), are the smallest subtype of EV, measuring 30–100 nm in diameter and surrounded by a phospholipid bilayer [54]. Exosomes are generated by exocytosis of multivesicular bodies and are released from cells into interstitial spaces and bodily fluids [55]. Secreted exosomes circulate in different fluids [53] and can be internalized by neighboring cells (in autocrine and paracrine communication) or by remote recipient cells (in endocrine communication) (Figure 4). They can also be transferred from one organism to another, thereby facilitating the exchange of genetic and epigenetic information between different organisms. Exosomes have been shown to be secreted in arthropod saliva [56] and transferred to vertebrate hosts, where they release their molecular contents, including exogenous lncRNAs.

LncRNAs in arthropods have not been well studied, and many of their roles are still unknown. They might be expressed in the host in response to pathogen infection or by the vector to counteract host defenses. It has been suggested that vector lncRNAs might be transported in salivary exosomes to the host, where they act as “sponges” of host miRNAs to disturb natural defense reactions (Figure 4); this hypothesis still requires experimental validation. Here we provide examples of recent studies on lncRNAs discovered in vectors or hosts upon pathogen transmission and their possible involvement in the tripartite vector–host–pathogen crosstalk.

Aedes mosquitoes are one of the most dangerous vectors known, transmitting several viruses such dengue (DENV) and Zika viruses [57]. Understanding the interaction between viruses and mosquito vectors and the factors involved in viral replication is essential for developing effective arbovirus control strategies. Consequently, several studies have focused on the dynamic interactions between *Aedes* mosquitoes and their vertebrate hosts with respect to ncRNAs, among other molecules. A comprehensive report of long intergenic ncRNAs encoded by the *Ae. aegypti* genome showed that a number of lncRNAs are differentially expressed in mosquitoes infected with dengue virus and might therefore be involved in DENV–mosquito interactions [58]. Another study with *Ae. albopictus* revealed a catalog of novel lncRNA transcripts in its genome and that their expression was altered upon infection with dengue and Zika viruses. The same study showed that depletion of certain lncRNAs resulted in the increased replication of dengue and Zika viruses, suggesting a potential role for lncRNAs in virus infection [59]. On the host side, it has also been shown that the vertebrate immune system can be modulated by lncRNAs to enhance virus survival during host infection. One well-studied example is the dengue virus, a single-stranded RNA virus belonging to the Flaviviridae family, which occurs as four antigenically distinct serotypes (DENV1–4) [60]. DENV infection causes intense immune activation in the host. Liver cells are a primary target for DENV infection, which often leads to direct or indirect liver damage and hepatitis as confirmed in biopsies or autopsies in some cases. To investigate the role of lncRNAs in DENV infection, Wang and colleagues analyzed the lncRNA expression profiles in L-02 human liver cells infected with DENV1 and DENV2 [61] and found four significantly regulated known lncRNAs in DENV1 and DENV2 infection groups compared to controls by RT-PCR. These findings were highly consistent with the RNA-sequencing analysis of the differentially expressed lncRNAs in the same samples. Consequently, the study suggested that host lncRNA expression might be regulated by invading pathogens [61].

In addition to Aedes, a recent study focused on Anopheles gambiae, the main vector of the most dangerous malaria parasite Plasmodium falciparum [62]. RNA-seq across multiple mosquito life stages and both genders revealed 2949 putative lncRNAs exhibiting low sequence conservation, with evidence that the secondary structural features of many were conserved. The study thus provided the basis for an expanded understanding of lncRNAs in dipterans and for future studies of ncRNAs in Anopheles [62]. This progress paves the way for malaria vector control strategies based on the repertoire of mosquito coding and non-coding genes.

LncRNAs have also been found in ticks, which are second only to mosquitos in terms of pathogen transmission to vertebrates. Tick salivary glands excrete molecules into the saliva in an apocrine manner, where whole pieces of cells are shed, and the cytoplasmic contents are excreted into the lumen of the salivary acinus. While tick salivary molecules have been widely investigated, only one study has reported putative lncRNAs in ticks that might target host defenses [63]. It has been suggested that tick lncRNAs compete with host mRNAs for host miRNA binding by acting as “sponge” molecules that inhibit host miRNA interactions with host mRNAs, thus affecting host homeostatic responses to tick feeding; again, this hypothesis requires experimental validation [64].

Overall, the functions of lncRNAs in arthropods, as well as whether they are transferred to the vertebrate host through exosomes, are still poorly understood. However, given the significant number of differentially expressed no ORF-containing transcripts observed in several organisms, the extensive transcriptional regulatory functions described for lncRNAs, and the potential host immunomodulation by parasite-derived lncRNAs, further investigation of these important RNA molecules in vector–host–pathogen networks is warranted.

## 5. Conclusions and Future Perspectives

Over the last decade, transcriptomics analyses have helped us to better understand the involvement of ncRNAs in host-parasite interactions [65]. ncRNAs have been widely studied to understand the host defense system and how it responds to infection via the immunoproteome [52,66]. Conversely, intracellular parasites regulate host responses by maintaining a close interaction with their hosts to ensure their persistence. In both these cases (host defenses and parasite success), several lncRNAs are believed to be involved in subverting cellular processes that lead to or regulate host defenses [67]. In this review, we discussed several features of lncRNAs including their classification, functions, and mechanisms of action and their possible roles in host-pathogen interactions. The roles of lncRNAs as miRNA sponges, guides, scaffolds, and decoys were highlighted, with examples. However, several lncRNA functions and/or their roles in different cellular processes remain to be determined. Moreover, several new classes of lncRNAs may yet be discovered. Current knowledge about lncRNAs suggests that host lncRNA responses are biologically important. Therefore, the exact roles of lncRNAs in host–pathogen interactions, and especially how pathogens modulate host lncRNAs to favor their survival, need intensive study. Newer techniques such as chromatin isolation by RNA purification (ChIRP-Seq), ribosome profiling, RNA structure mapping, crosslinking immunoprecipitation (CLIP), targeted genome engineering by clustered regularly interspaced short palindromic repeats (CRISPR), and phylogenetic lineage tracing are likely to deepen our insight into the functions and biogenesis of lncRNAs. These techniques will allow us to understand lncRNAs interaction with other RNA types or proteins at the vector, pathogen, and host levels. As such, future work on the molecular basis of host responses to parasitic interactions should also focus on lncRNAs to provide new insights into the control and diagnosis of parasitic infections.

## Abbreviations

ChIRP-Seq: chromatin isolation by RNA purification; CRISPR: clustered regularly interspaced short palindromic repeats; COLDAIR: cold-assisted intronic non-coding RNA; ceRNAs: competing endogenous RNA; CLIP: crosslinking immunoprecipitation; ERs: enhancer regions; eRNA: enhancer RNA; FIRRE: functional intergenic repeating RNA element; H19: H19 imprinted maternally expressed transcript; HOTAIR: HOX transcript antisense RNA; HOTTIP: HOXA distal transcript antisense RNA; lincRNAs: long intervening non-coding RNAs; lncRNAs: long non-coding RNAs; MALAT1: metastasis-associated lung adenocarcinoma transcript 1; miRNAs: microRNAs; mRNAs: messenger RNAs; NAT: natural antisense transcripts; ORFs: open reading frames; piRNAs: p-element induced wimpy interacting RNAs; RNaseP: ribonuclease P; RNA: ribonucleic acid; RNP: ribonucleoprotein; rRNAs: ribosomal RNAs; RNAi: RNA interference; siRNAs: small-interfering RNAs; sncRNAs: small non-coding RNAs; TEs: transposable elements
